# Supplementary material for: Seroprevalence of SARS-CoV-2 in Client-Owned Cats from Portugal
Source: Vet Sci. 2022 Jul 16;9(7):363. doi: 10.3390/vetsci9070363 (PMC9315516; doi:10.3390/vetsci9070363)
Supplement: Supplementary file 1 [file vetsci-09-00363-s001.zip › vetsci-1770402-supplementary.pdf]

Sample	OD (mean two replicates)	S/P%
1	0.1241	0.563043849
2	0.1348	1.475857362
3	0.1384	1.782972189
4	0.1196	0.1791503156
5	0.1142	0.2815219245
6	0.1099	0.6483535232
7	0.239	10.36512541
8	0.3209	17.35198772
9	0.1184	0.076778707
10	0.1402	1.936529602
11	0.1349	1.48438833
12	0.1336	1.373485753
13	0.1396	1.885343798
14	0.1585	3.497696639
15	0.1301	1.074901894
16	0.1356	1.544105102
17	0.1231	0.477734175
18	0.1287	0.95546835
19	0.1289	0.972530285
20	0.1307	1.126087698
21	0.1387	1.808565091
22	0.1289	0.972530285
23	0.1495	2.729909572
24	0.1386	1.800034124
25	0.1385	1.791503156
26	0.1404	1.953591537
27	0.1357	1.552636069
29	0.1405	1.962122505
29	0.1585	3.497696639
30	0.1389	1.825627026
31	0.136	1.578228971
32	0.1604	3.65978502
33	0.1507	2.832281181
34	0.1514	2.891997953
35	0.2353	10.04947961
36	0.153	3.028493431
37	0.1462	2.448387647
38	0.2347	9.998293807
39	0.15	2.772564409
40	0.1335	1.364954786
41	0.1432	2.192458625
42	0.2334	9.88739123
43	0.1514	2.891997953
44	0.2387	10.3395325
45	0.141	2.004777342

46	0.1459	2.422794745
47	0.1324	1.271114144
48	0.1574	3.403855997
49	0.123	0.469203208
50	0.1178	0.025592902
51	0.1219	0.375362566
52	0.1291	0.98959222
53	0.1217	0.358300631
54	0.125	0.639822556
55	0.1317	1.211397372
56	0.1387	1.808565091
57	0.4761	30.59204914
58	0.1375	1.706193482
59	0.1419	2.081556048
60	0.1845	5.715748166
61	0.1492	2.70431667
62	0.1279	0.887220611
63	0.1365	1.620883808
64	0.1331	1.330830916
65	0.1437	2.235113462
66	0.1339	1.399078656
67	0.1703	4.504350793
68	0.1416	2.055963146
69	0.1434	2.20952056
70	0.1427	2.149803788
71	0.1691	4.401979184
72	0.1495	2.729909572
73	0.1767	5.050332708
74	0.1361	1.586759939
75	0.2077	7.694932605
76	0.1431	2.183927657
77	0.1754	4.939430131
78	0.1271	0.818972872
79	0.1599	3.617130183
80	0.1532	3.045555366
81	0.4365	27.21378604
82	0.1408	1.987715407
83	0.1647	4.026616618
84	0.1466	2.482511517
85	0.1713	4.589660467
86	1.7203	136.7343457
87	0.1494	2.721378604
88	0.1369	1.655007678
89	0.149	2.687254735
90	0.1236	0.520389012
91	0.1449	2.337485071

92	0.2063	7.575499062
93	0.1275	0.463747436
94	0.131	0.775885133
95	0.1436	1.899580844
96	0.1563	3.032194774
97	0.1501	2.479265139
98	0.1358	1.20395969
99	0.1329	0.945331312
100	0.136	1.221796129
101	0.1734	4.557210381
102	0.1506	2.523856238
103	0.1309	0.766966913
104	0.1211	-0.107018639
105	0.1578	3.165968073
106	0.1327	0.927494872
107	0.1325	0.909658432
108	0.1459	2.104699902
109	0.1363	1.248550789
110	0.1311	0.784803353
111	0.1478	2.27414608
112	0.1322	0.882903772
113	0.1337	1.016677071
114	0.1906	6.091144208
115	0.1419	1.747971105
116	0.1067	-1.391242308
117	0.1261	0.338892357
118	0.1279	0.499420316
119	0.1284	0.544011415
120	0.1543	2.853830375
121	0.1374	1.346651208
122	0.1207	-0.142691519
123	0.136	1.221796129
124	0.1332	0.972085972
125	0.13	0.686702934
126	0.1478	2.27414608
127	0.1817	5.297422634
128	0.1213	-0.089182199
129	0.1298	0.668866494
130	0.2296	9.569249978
131	0.1363	1.248550789
132	0.1436	1.899580844
133	0.212	7.999643271
134	0.1364	1.257469009
135	1.7498	145.1440293
136	0.2228	8.962811023
137	0.1368	1.293141889

138	0.1592	3.290823152
139	0.1418	1.80980938
140	0.1557	3.29835082
141	0.1385	1.45641465
142	0.1413	1.756264725
143	0.1405	1.670593275
144	0.1534	3.052045406
145	0.1384	1.445706
146	0,1874	6.69308203
147	0,1541	3.127007925
148	0,1669	4.497751124
149	2.3851	242.0432641
150	0,1477	2.441636325
151	0.1386	1.467123581
152	0.1487	2.548725637
153	0.4932	39.44099379
154	0.16	3.758834868
155	0.3004	18.79417434
156	0.1485	2.527307775
157	0.1339	0.963803812
158	0.1379	1.392161062
159	0.1373	1.327907475
160	0.1406	1.681302206
161	0.1362	1.210109231
162	0.1699	4.819019062
163	0.1592	3.673163418
164	0.1632	4.101520668
165	0.1432	1.959734419
166	0.1549	3.212679375
167	0.152	2.902120368
168	0.1516	2.859284643
169	0.1269	0.214178625
170	0.1617	3.9408867
171	0.1733	5.183122724
172	0.2268	10.91240094
173	0.1664	4.444206468
174	0.2379	12.10109231
175	0.1487	2.548725637
176	0.1649	4.283572499
